# Effects of different protein levels on growth performance and stress parameters in beef calves under heat stress

**DOI:** 10.1038/s41598-022-09982-4

**Published:** 2022-05-17

**Authors:** Won Seob Kim, Jalil Ghassemi Nejad, Dong Qiao Peng, Yong Ho Jo, Jongkyoo Kim, Hong Gu Lee

**Affiliations:** 1 Department of Animal Science and Technology, Sanghuh College of Life Sciences, Konkuk University, Seoul, 05029 Korea; 2 Animal Science and Food Science and Human Nutrition, Michigan State University, East Lansing, MI 48824 USA; 3 Present Address: Department of Animal Science, Michigan State University, East Lansing, MI 48824 USA

**Keywords:** Zoology, Climate sciences

## Abstract

This study investigated the effects of dietary protein levels under various heat stress (HS) conditions on the growth performance and stress parameters in Korean native beef calves. Male calves (n = 40; initial BW = 202.2 ± 3.31 kg) were randomly assigned to climatic-controlled chambers with 3 × 3 factorial arrangements. Calves were assigned into three dietary protein levels (low protein; LP = 12.5%, medium protein; MP = 15%, and high protein; HP = 17.5%) and three HS levels [mild: temperature-humidity index (THI) = 74 to 76, moderate: THI = 81 to 83, and severe: THI = 89 to 91] with control (threshold: THI = 70 to 73 and dietary protein level 12.5%). The calves were subjected to ambient temperature (22 °C) for 7 days and subsequently to the temperature and humidity corresponding to the target THI level for 21 days. The data were analyzed using the repeated-measures analysis by the GLM procedure of SAS. As a result, average daily gain (ADG) was decreased (*P* < 0.05) under severe HS level compared to the mild and moderate HS stress levels. However, HP increased ADG (*P* < 0.05) than moderate levels (LP) and severe levels (LP and MP). Under different HS levels (mild, moderate, and severe), HR, RT, and blood cortisol were increased (*P* < 0.05) compared to a threshold level, but no differences were observed in the parameters among various protein levels. Varied HS levels decreased the levels of blood glucose, NEFA, and amino acids (AAs) (lysine and glutamic acid) compared to a threshold (*P* < 0.05). But, the HP group resulted in increased (*P* < 0.05) levels of blood glucose, NEFA, and AAs (lysine and glutamic acid) compared to LP and MP groups under severe HS stress. The expression level of the HSP70 gene in peripheral blood mononuclear cell (PBMC) and hair follicles was increased (*P* < 0.05) following an increase in moderate and severe HS levels. Also, HSP70 gene expression in the HP group was decreased (*P* < 0.05) compared with LP and MP groups under intense HS level. Overall, HS in Korean native beef calves exhibited negative effects on ADG, blood glucose, NEFA, and AA profile. However, 17.5% of dietary protein (HP) could compensate for the growth of heat-exposed Korean native beef calves through the regulation of homeostasis by protein and energy metabolism. Also, it was evident that adequate protein (HP) is used as a major nutrient for HSP70 synthesis in PBMC and hair follicles causing, a boost in the immune system of heat-exposed Korean native beef calves.

## Introduction

The drastic ambient temperature and humidity increase can create multiple issues in animal agriculture. Temperature above the animals' thermoneutral zone decreases farm profitability and influences animals' physiology^1,2^. HS can be detrimental to lactation, growth, and reproduction in most livestock animals^3^. In ruminant, the impact of the phenomenon will affect not only the dairy industry ($1.2 billion), but also the beef industry ($369 million) resulting in serious economic losses^4^. Heat stress induces behavioral and metabolic changes in cattle that are intended to maintain homeothermy, often at the expense of decreased productivity and profitability^5^. Various responses to HS have been reported, including reduced dry matter intake (DMI), activity, productivity, and increased metabolic rate. Among these HS feedback, reduced feed intake is the first noticeable sign of HS, presumably an evolutionary strategy to reduce the "heat increment" of feeding^6,7^. This leads to dystrophia and is known to reduce production in growing cattle^8^.

Previous studies suggested management strategies to reduce the unfavorable outcomes from HS, such as physical and genetic modification and improved nutritional management^5,9^. Genetical improvement against HS can be achieved by introducing thermostable breeds. *Bos indicus* are more resistant to heat than *Bos taurus* because they have physiological and cellular thermotolerance^10,11^. Nutritional modifications also have suggested compensating for the adverse effects of HS. Overall intake usually reduces during hot weather, and it may lead to reverse growth. In order to offset the reduced feed intake and increased nutrient requirements during HS, diet reformulation is required^12^. In growing calves, protein is an essential nutrient to maintain skeletal muscle growth^13^. In calves in a hot environment, protein supplementation levels need to be increased to compensate for reduced feed intake^13^.

We hypothesize that a greater protein level in the diet of heat-stressed growing calves would improve their growth performance according to various responses in body mechanisms such as homeostasis, gluconeogenesis, and amino acid (AA) synthesis using dietary protein level. In addition, limited information is available regarding the dietary protein level of beef calves during the HS situation. Despite that NASEM^13^ states guidance of specification managements under HS, it lacks details. Therefore, the objective of the current study was to (1) demonstrate the different dietary protein levels under various HS conditions on growth performance and stress parameters (2) to provide a guide line for dietary protein supplementation under the HS condition in Korean native beef calves.

## Material and methods

### Animals, management conditions and treatments

All procedures involving animals were approved by the Institutional Animal Care and Use Committee (IACUC) of Konkuk University (Approval No: KU18178), and animal care was performed according to the committee’s guidelines and as per ARRIVE Guidelines/Checklist. For the selection criteria, the hair follicles samples from all animals (forty-six) were collected for DNA extraction and the heat shock protein 70 (HSP70) genotype was analysed based on polymerase chain reaction-restriction fragment length polymorphism (PCR–RFLP) before starting the experiment. Forty calves were selected among forty-six calves based on HSP70 genotyping^14^ in order to homogenize heat tolerance characteristics. Forty male calves (body weight of 202.2 ± 3.31 kg) were randomly allotted to ten dietary treatments with four replications per treatment. A completely randomized design using a 3 × 3 factorial arrangements with 3 dietary protein levels (low protein; LP = 12.5%, medium protein; MP = 15%, high protein; HP = 17.5%) and 3 stress levels [mild: temperature-humidity index (THI) = 74 to 76, moderate: THI = 81 to 83, severe: THI = 89 to 91] was conducted. A control group (threshold) was assigned at a THI level of 70–73 with a dietary CP level of 12.5%. The calves were exposed to the ambient temperature of 22 °C for 7 days (Thermoneutral: TN), including one week of the adaptation period prior to the start of the experiment. Following that, the temperature and RH in the chambers were raised to each THI level for 21 days (HS). Three THI treatments were categorized as mild (26–28 °C, 60% RH: THI = 74 to 76), moderate (29–31 °C, 80% RH: THI = 81 to 83), and severe (32–34 °C, 80% RH: THI = 89 to 91) stress levels with a control group (threshold, 22–24 °C, 60% RH: THI = 70 to 73)^15^.

**Figure 1:**
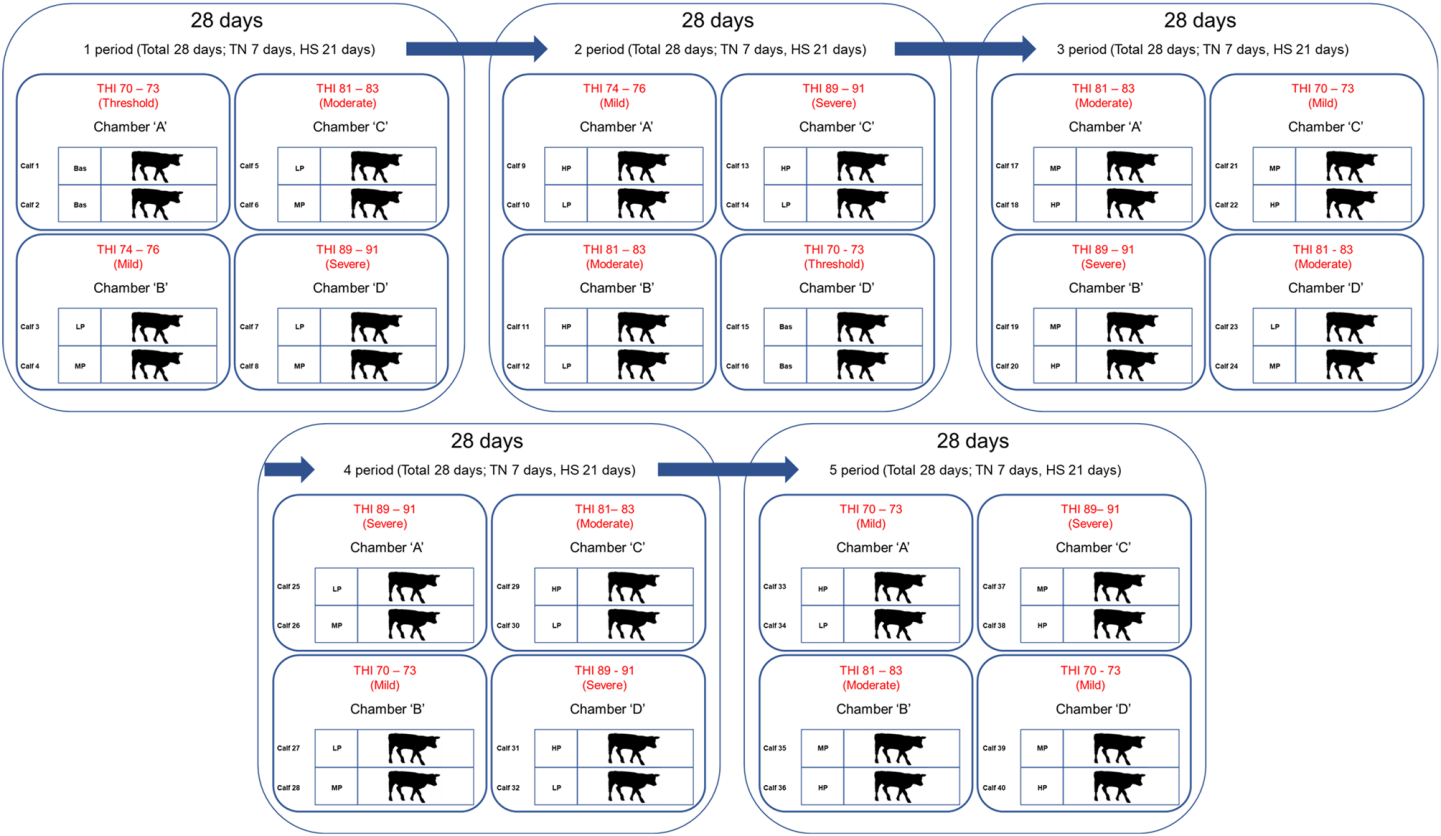
Experiment design. In our study the forty calves were divided to 8 calves per period (total five periods) to assign to one of the four chambers (two calves per chamber) with the respective protein treatment being assigned to the calves in the climatic chamber containing tie-stall stanchions.

In our study the forty calves were divided to 8 calves per period (total five periods) to assign to one of the four chambers (two calves per chamber) with the respective protein treatment being assigned to the calves in the climatic chamber containing tie-stall stanchions (Fig. 1). The size of each climatic chamber was 2.5 m × 2.5 m × 3 m (length, width, and height, respectively). The chambers were designed and programmed to operate over a temperature range of 18–34 °C and a humidity range of 60–100% RH. The temperature and RH in a chamber were controlled by regulators to maintain THI for each treatment from 0900 to 1900 h. During the night-time (from 1900 to 0900 h), THI was maintained under 68, which does not influence the physiological parameters of the animals^16^. The study included four different THI groups and was designed to consist of four levels of dry bulb temperature (22–24 °C, 26–28 °C, 29–31 °C, 32–34 °C) and two levels of RH (60 and 80%). Temperature and RH inside the chamber were recorded at 1 s intervals of using two sensors (SHT7x, Sensirion AG, Staefa, Switzerland). The THI was calculated using the dry bulb temperature (T_db_, °C) and RH using the following formula^17^:

$$ THI = \left( {1.8 \times T_{db} + 32} \right){-}\left[ {\left( {0.55{-}0.0055 \times RH} \right) \times \left( {1.8 \times T_{db} - 26.8} \right)} \right] $$

Animals were offered feed and water individually. The diets used in this study were composed of 40% roughage (*Phleum pratense* L.) and 60% concentrate. The feed was weighed and offered twice a day at 0900 and 1700 h (Table 1). Feed and water intake were measured on daily basis (0900 h). The chemical compositions of the feed are shown in Table 2. The BW of each animal was measured at d0 and d28 after trial initiation before the morning feed delivery (0800 h).

**Table 1:** Ingredient composition of experimental diets.

	Threshold	Mild			Moderate			Severe		
	Bas	LP	MP	HP	LP	MP	HP	LP	MP	HP
**Ingredient, % of DM**										
Corn grain, steamed flaked	21.1	28.5	28.3	28.8	29.8	29.5	28.7	29.9	29.2	29.8
Soybean meal	8.9	7.9	14.5	27.5	10.1	18.2	27.2	10.1	18.3	27.1
Wheat bran	21.9	14.1	3.7	1.5	9.9	2.8	0.3	9.8	2.8	0.2
Corn germ meal	6.1	7.5	11.5	0.2	8.2	7.5	1.8	8.2	7.7	0.9
Timothy hay	40	40	40	40	40	40	40	40	40	40
Limestone	1.9	1.9	1.9	1.9	1.9	1.9	1.9	1.9	1.9	1.9
Vit and mineral premix*	0.1	0.1	0.1	0.1	0.1	0.1	0.1	0.1	0.1	0.1

*The premix contained (% as-is basis): trace mineral mix, 0.86; MgO (56% Mg), 8.0; NaCl, 6.4; vitamin ADE premix, 37.2; selenium premix, 0.07; and dry corn distillers grains with soluble, 45.7%; Ca, 12.1%; P, 0.41%; Mg, 4.59%; K, 0.44%; S, 0.39%; Se, 6.91 mg/kg; Cu, 383 mg/kg; Zn, 884 mg/kg; Fe, 216 mg/kg, vitamin A, 300,000 IU/kg, vitamin D, 85,000 IU/kg and vitamin E, 2,300 IU/kg.

THI = Temperature humidity index, THI = (1.8 × Tdb + 32) – [(0.55 – 0.0055 × RH) × (1.8 × Tdb − 26.8).

Threshold = THI 70 to 73, Mild = THI 74 to 76, Moderate = THI 81 to 83, Severe = THI 89 to 91.

LP = Low protein (12.5%), MP = Medium protein (15%), HP = High protein (17.5%).

**Table 2:** The chemical composition of experimental diets provided to the Korean native beef calves.

	Threshold	Mild			Moderate			Severe		
	Bas	LP	MP	HP	LP	MP	HP	LP	MP	HP
**Analyzed composition, % of DM**										
CP	12.48	12.47	14.89	17.39	12.52	15.04	17.54	12.59	15.10	17.45
RDP^1^	5.96	5.46	6.18	7.04	5.30	6.13	7.06	5.32	6.17	6.95
RUP^1^	6.39	7.01	8.71	10.35	7.22	8.91	10.48	7.27	8.93	10.50
TDN^1^	70.44	72.17	72.88	73.20	72.68	73.17	73.27	72.71	73.12	73.45
TDN^1^ (kg)	3.63	3.06	3.09	3.10	3.03	3.05	3.06	2.78	2.79	2.81
NDF	40.62	38.48	37.37	35.02	37.69	36.37	35.09	37.66	36.45	34.78
ADF	20.70	19.96	19.73	19.48	19.75	19.56	19.47	19.74	19.59	19.38
Fat	3.83	3.81	4.36	4.05	3.93	4.20	4.16	3.93	4.22	4.08
Ash	5.71	5.37	5.40	5.65	5.33	5.44	5.64	5.33	5.45	5.60
Ca	0.94	0.93	0.94	0.98	0.93	0.95	0.97	0.93	0.95	0.97
P	0.49	0.44	0.42	0.36	0.42	0.39	0.37	0.42	0.39	0.36
ME^1^ (Mcal/kg)	2.55	2.61	2.63	2.65	2.63	2.64	2.65	2.63	2.64	2.65
NEm^1^ (Mcal/kg)	1.64	1.69	1.72	1.73	1.71	1.73	1.73	1.71	1.72	1.73
NEg^1^ (Mcal/kg)	1.04	1.08	1.10	1.11	1.09	1.11	1.11	1.09	1.11	1.11

^1^Values were estimated using NRC (2016).

THI = Temperature humidity index, THI = (1.8 × Tdb + 32) – [(0.55 – 0.0055 × RH) × (1.8 × Tdb − 26.8).

Threshold = THI 70 to 73, Mild = THI 74 to 76, Moderate = THI 81 to 83, Severe = THI 89 to 91.

LP = Low protein (12.5%), MP = Medium protein (15%), HP = High protein (17.5%).

### Chemical analysis

Dry matter (DM; method 930.15), crude protein (CP; method 984.13), crude ash (method 942.05), crude fat (method 920.39), ADF (method 973.18) were analyzed according to AOAC^18^. The NDF content was determined according to the procedure of Van Soest et al.^19^. The level of Ca and P were measured inductively coupled plasma spectroscopy (method 945.46)^18^. While DM was determined by drying ground diets in a vacuum oven at 100 °C overnight, crude ash content was measured by incineration at 550 °C overnight in a muffle furnace (KMF-500, Lab Corporation, Seoul, South Korea). In order to measure CP contents, total nitrogen content in each diet was measured using the Kjeltec™ System (Kjeltec™ 2400, FOSS, Denmark), and the final CP content was calculated as nitrogen × 6.25. Crude fat content was analyzed using the ether extraction system (ANKOMXT15 Extractor, ANKOM Technology, Macedon, NY). Mineral content was determined by inductively coupled plasma optical emission spectrometry (ICP-OES, Thermo-Fisher Scientific, Waltham, MA). The AA composition in each diet was determined using the AA analyzer (Beckman 6300, Beckman, Fullerton, CA) (Table 3).

**Table 3:** Amino acids (AA) concentration (%) of experimental diets.

	Threshold	Mild			Moderate			Severe			SEM	*P* value
	Bas	LP	MP	HP	LP	MP	HP	LP	MP	HP		
**Analyzed composition, % of DM**												
Lysine	0.34^b^	0.31^b^	0.67^ab^	1.42^a^	0.27^b^	0.81^ab^	1.67^a^	0.22^b^	0.52^ab^	1.52^a^	0.058	< 0.01
Methionine	0.21^b^	0.24^b^	0.31^ab^	0.52^a^	0.22^b^	0.44^a^	0.51^a^	0.19^b^	0.42^a^	0.41^a^	0.067	< 0.01
Aspartic acid	0.62	0.77	0.81	0.72	0.81	0.71	0.68	0.62	0.68	0.61	0.041	0.13
Serine	0.42	0.57	0.62	0.45	0.42	0.62	0.54	0.52	0.58	0.47	0.028	0.65
Glutamic acid	2.14^c^	2.28^c^	2.81^b^	3.51^a^	2.12^c^	2.77^b^	3.62^a^	2.12^c^	2.92^b^	3.42^a^	0.072	< 0.01
Glycine	0.72	0.67	0.71	0.82	0.55	0.62	0.77	0.57	0.62	0.85	0.048	0.72
Alanine	1.13	0.92	1.12	1.14	0.81	1.09	1.02	0.74	1.11	1.41	0.082	0.69
Valine	0.82^b^	0.85^b^	0.91^b^	1.32^a^	0.71^b^	0.82^b^	1.21^a^	0.92^b^	0.82^b^	1.42^a^	0.032	< 0.01
Isoleucine	0.57^b^	0.62^b^	0.91^a^	0.82^a^	0.55^b^	0.92^a^	0.71^ab^	0.52^b^	0.97^a^	0.97^a^	0.057	< 0.01
Leucine	1.92^b^	1.82^b^	1.89^b^	2.42^a^	1.71^b^	1.67^b^	2.62^a^	1.87^b^	1.97^b^	2.32^a^	0.081	< 0.01
Tyrosine	0.42	0.32	0.47	0.54	0.42	0.55	0.31	0.52	0.37	0.33	0.074	0.82
Phenylalanine	0.65	0.72	0.62	0.67	0.55	0.71	0.77	0.91	0.71	0.58	0.092	0.51
Histidine	0.45	0.42	0.57	0.55	0.42	0.52	0.42	0.37	0.52	0.47	0.074	0.84
Arginine	0.92	0.82	1.12	0.92	0.91	1.09	0.81	0.97	0.88	0.82	0.078	0.64
Tryptophan	0.14	0.11	0.17	0.15	0.21	0.07	0.09	0.11	0.21	0.09	0.001	0.67
Cystine	0.24	0.32	0.33	0.42	0.44	0.42	0.37	0.42	0.41	0.38	0.081	0.75
Proline	0.92	0.52	0.87	0.71	0.62	0.51	0.62	0.41	0.62	0.59	0.031	0.82

THI = Temperature humidity index, THI = (1.8 × Tdb + 32) – [(0.55 – 0.0055 × RH) × (1.8 × Tdb − 26.8).

Threshold = THI 70 to 73, Mild = THI 74 to 76, Moderate = THI 81 to 83, Severe = THI 89 to 91.

LP = Low protein (12.5%), MP = Medium protein (15%), HP = High protein (17.5%).

^a, b^Values with different superscripts in the same row differ significantly at *P* < 0.05.

### Physiological parameters under heat stress

The physiological parameters of heat-stressed calves, including heart rate (HR) and rectal temperature (RT), were measured every three days (1400 h)^20^. The HR, expressed in beats per minute (BPM), was obtained using a stethoscope (TS-DIA01002, Tenso Medical Instrument Co., Zhejiang, China) placed directly onto the left thoracic region under one of the auscultation foci for one minute. The RT was measured after checked HR with a large animal clinical thermometer (TES-1300 Thermometer, E&E PROCESS Instrument Co., Vaughan, ON, Canada) that was inserted to a depth of 3 cm into the animal’s rectum and held in contact with the mucosa for one minute.

### Blood parameters under heat stress

Blood samples were collected every three days (d1, 4, 7, 10, 13, 16, 19, 22, 25, and 28) at 1400 h from jugular venepuncture using non-heparinized vacutainers (20 mL; Becton–Dickinson, Belliver Industrial Estate, PL6 7BP, Plymouth, UK) and ethylenediaminetetraacetic acid-treated vacutainers (4 mL; Becton–Dickinson, Franklin Lakes, NJ, USA)^21^. Serum samples were obtained from blood after centrifugation at 2700× *g* at 4 °C for 15 min. Serum was transferred to a 1.5 mL tube (Eppendorf AG, Hamburg, Germany) and kept at − 80 °C until further analysis. Serum was analyzed for glucose (Fuji Dri-Chem Slide Glu-PIII, Fuji Film Corp., Tokyo, Japan) and blood urea nitrogen (BUN, Fuji Dri-Chem Slide BUN-PIIIs, Fuji Film Corp., Tokyo, Japan) using DRI CHEM 7000i biochemistry analyzer (Fuji Film, Tokyo, Japan). Non-esterified fatty acids (NEFA) was analyzed using Roche Free Fatty Acids kits (Roche, Mannheim, Germany) with HITACHI 7600 chemistry autoanalyzer (Hitachi, Tokyo, Japan). Blood cortisol was determined using a commercial Bovine ELISA test kit (Life Diagnostics, Inc, West Chester, PA, USA). Plasma was deproteinized with 10% sulfosalicylic acid for analysis of AA profiles using the AA analyzer (Sykam S433, Sykam GmbH, Germany).

### Sampling and isolation of peripheral blood mononuclear cells

The PBMC isolation from collected blood was conducted within 8 h of the sample collection. Density gradient centrifugation was used to separate PBMCs from the whole blood. The whole blood was diluted 1:1 with 1 × PBS (Hyclone, Laboratories, INC., Logan, UT, USA) and layered gently over Histopaque-1077 (Sigma-Aldrich Inc., St. Louis, MO, USA). All the PBMC isolation steps were performed at room temperature, following the manufacturer's instructions^22^.

### Hair follicle collection and storage

The hair follicle samplings were conducted as previously reported by Kim et al.^15^. Hair follicles were collected from the tails of each calf every three days at 1400 h. Approximately 25–30 strands of hair were pulled from the tail head of calves. Hairs were grasped as close to the skin as possible and then rapidly pulled out. The hair follicles were washed using DEPC-treated water. The hair follicles were then placed into a 5 mL specimen jar filled with RNAlater™ (Ambion, Austin, TX) for total RNA extraction. Total RNA was extracted from the hair follicles on the day of collection and stored at room temperature for 14 days in RNAlater™ (Ambion, Austin, TX, USA). The hair segments, containing the hair follicle (1 cm from bottom), were cut, and transferred into a 2 ml microcentrifuge tube contained 1 ml of TRIzol™.

### Total RNA extraction and real-time PCR analysis

Total RNA was extracted from PBMCs and hair follicles using TRIzol™ reagent (Invitrogen, Carlsbad, CA, USA) according to the manufacturer’s instructions^22^. The RNA quality and quantity of isolated were measured using an ND-1000 spectrophotometer (NanoDrop Technologies, Wilmington, DE, USA). The A260/280 ratios of all RNA samples were greater concentration than 1.8. The RNA quality was assessed using an RNA 6000 Nano Lab Chip kit (Agilent, Palo Alto, CA, USA). The RNA integrity number (RIN) was confirmed in a Bioanalyzer 2100 (Agilent, Palo Alto, CA, USA) to determine whether the purified total RNA could be used in real-time PCR. The average RIN of PBMCs was 8.4 (7.8–9.4). The RNA samples were stored at − 70 °C until analysis. The first-strand cDNA was synthesized using RNA (1 μg) and an iScript cDNA synthesis kit (Bio Rad, Hercules, CA, USA). The expression of HSP70 gene in PBMCs was analyzed by real-time quantitative PCR (RT qPCR) amplification with SYBR- Green® as described previously^23^. All reactions were performed in triplicate and a total reaction volume of 20 μL per well in a 96-well plate using a Chromo4™ four-color real-time detector (MJ Research, Waltham, MA, USA). The reaction mixture contained 100 ng of cDNA, 10 μL of 2 × SYBR Green PCR master mix (Bio-Rad) and 0.6 μL of primers at 10 μM (Bioneer, Daejeon, South Korea) in autoclaved water. The thermal cycling conditions were as follows: initial incubation at 95 °C for 3 min followed by 40 cycles of denaturation at 95 °C for 10 s, annealing at 60 °C for 30 s, and extension at 72 °C for 30 s, after which the samples were heated at 95 °C for 10 s, cooled to 65 °C for 5 s and then heated to 95 °C at a rate of 0.5 °C/s. The results were monitored using post-PCR melt curve analysis of amplification reactions (in triplicate from all samples) and sequencing amplification products. Primers were designed using the National Center for Biotechnology Information Primer-BLAST (Table 4). The threshold cycles for each sample were normalized to housekeeping genes (GAPDH, RPS15A, and B2M)^24^, and the relative expression of the target gene was quantified as the fold change of expression of the target gene relative to the expression of the thermoneutral control according to the 2 − ΔΔrCT method^25^. The coefficient of variation of the housekeeping gene was checked before calculating of the results to ensure that it did not exceed 5%.

**Table 4:** Primer sequences, lengths, and accession numbers in bovine.

Gene	Accession number	Sequence	Length (bp)
HSP70	U09861	F: TACGTGGCCTTCACCGATAC R: GTCGTTGATGACGCGGAAAG	171
GAPDH	NM_001034034.2	F: GGCAAGGTCATCCCTGAG R: GCAGGTCAGATCCACAACAG	166
RPS15A	NM_001037443.2	F: CCGTGCTCCAAAGTCATCGT R: GGGAGCAGGTTATTCTGCCA	200
B2M	NM_173893.3	F: GACACCCACCAGAAGATGGA R: CAGGTCTGACTGCTCCGATT	125

*HSP* heat shock protein, *GAPDH* glyceraldehyde-3-phosphate dehydrogenase, *RPS15A* ribosomal protein S15a, *B2M* beta-2-microlobulin.

### DNA extraction from hair follicle for genotype of HSP70

The DNA was extracted from the hair follicle using the manual method^26^. Hair follicles were cut off 5–10 mm of 20 hairpieces and placed into a 1.5 mL tube. The 300 μL tissue lysis (TL) buffer contained 1 M Tris–Cl pH 8.0, 0.5 M ethylenediaminetetraacetic acid (EDTA) pH 8.0, 5 M NaCl, 10% sodium dodecyl sulfate, and distilled water were added and mixed by inverting. Then, 5 μL proteinase K was added (20 mg/mL, G-spin Total DNA extraction Kit, iNtRON Biotechnology, Seongnam, South Korea), followed by incubation at 56 °C overnight. Following that, 300 μL protein precipitation solution was added (7 M Ammonium acetate, Tech & Innovation, Seongnam, South Korea) and centrifuged at 13,000 rpm 20 °C for 15 min. After this, 600 μL of supernatant was transferred to a new 1.5 mL tube and incubated at 65 °C for 1 h after 10 μL RNase solution was added (10 mg/mL, G-spin Total DNA extraction Kit, iNtRON Biotechnology, South Korea). Subsequently, 600 μL was added into the tube and centrifuged again (13,000 rpm, 20 °C for 15 min). The solution was divided into two other 1.5 mL tubes, with each tube for 600 μL, and this was followed by adding 600 μL isopropanol to each tube, inverting the sample, and incubating at − 20 °C for 1 h. After that, it was centrifuged at 13,000 rpm, 4 °C for 10 min, then the supernatant was discarded, and 300 μL 70% EtOH was added to wash the DNA extraction. Finally, the sample was centrifuged at 13,000 rpm, 4 °C for 5 min, and the supernatant was discarded. The pellet was dried at room temperature for 1 h, then 50 μL Tris–EDTA (TE) buffer was added (1× TE buffer, pH 8.0, Tech & Innovation, South Korea) for storage.

### Polymerase chain reaction and restricted fragment length polymorphisms

Primer sequences for the target gene HSP70 were 5′-GCCAGGAAACCAGAGACAGA-3′ (forward), 3′-CCTACGCAGGAGTAG-3′ (reverse), which was referenced from GenBank (accession number M98823.1). HSP70 gene was amplified by 2× Taq polymerase chain reaction (PCR) premix kit (Solgent Co. Ltd, Daejeon, South Korea) followed by using the T100 96-well Thermal Cycler (Bio-Rad Laboratories Inc, Seoul, South Korea): 95 °C for 2 min, 35 cycles of 20 s at 95 °C, 40 s at 56 °C, 1 min for 72 °C, and finally 5 min at 72 °C. The product of PCR was digested by BslI (New England Biolabs, Pickering, ON, Canada) for 3 h, in which the C allele was cut into 895-bp and 1128-bp fragments^14^. Then, the type of HSP70 was shown by the separation of electrophoresis according to a 2% agarose gel.

### Statistical analysis

THI data, growth performance, physiological parameters, blood parameters, and HSP gene expression were analyzed using repeated-measures analysis and the GLM procedure in SAS version 9.4 (SAS Institute Inc., Cary, NC, USA). The model was as follows:

$$ {\text{Y}}_{ijk} =\upmu + \upalpha _{i} +\upbeta _{j} + (\upalpha \upbeta )_{ij} +\upgamma \left( \upalpha  \right)_{ik} +\upvarepsilon _{ijk} $$

where Y_*ijk*_ is the observation of calf *k* for the given treatment *i*, *j*, μ is the overall mean, α_*i*_ is the fixed effect of treatment *i* (HS: mild, moderate, and severe level), β_*j*_ is the fixed effect of treatment *j* (protein level: LP, MP, and HP), (αβ)_*ij*_ is the interaction effect, γ(α)_*ik*_ is the random effect of calf *k* nested in treatment *i*, *j* and ε_*ijk*_ is the residual effect. Calf identity was included in the model as a random effect. The subject of the REPEATED statement was the effect of the calf. Preplanned orthogonal contrasts were used to calculate the linear effects (dietary protein and HS level) and conducted to test the effects of dietary protein (Control vs. 9 experimental diets), dietary protein levels (12.5, 15, and 17%), HS levels (mild, moderate, and severe), and the interaction between dietary protein level and HS level. A Tukey's honest significant difference (HSD) test was performed for mean comparisons. The covariance structures (autoregressive order 1, unstructured and compound symmetry) for the repeated measures model were tested and the structure that best fit the model was chosen based on the smallest value of Schwarz's Bayesian information criterion. The first day of sampling in each THI group was included as a covariate to correct the means. The covariate factor was included in the model when appropriate but was removed from the model when it was insignificant. Data are presented as least square means and associated standard errors. We conducted a post hoc power analysis using G*Power (version 3.1.9.7, University of Düsseldorf, Düsseldorf, Germany) to verify the analysis of the difference between groups in this study. The post hoc power analysis was applied with α = 0.05, sample size = 40, and effect size = 0.74. The power of analysis (1-β) for the difference among the groups was 0.83. Differences were considered statistically significant if the *P* value was less than 0.05. Means with *P* values between 0.05 and 0.10 reflected a tendency to differ.

## Results

### Identification of polymorphisms

**Figure 2:**
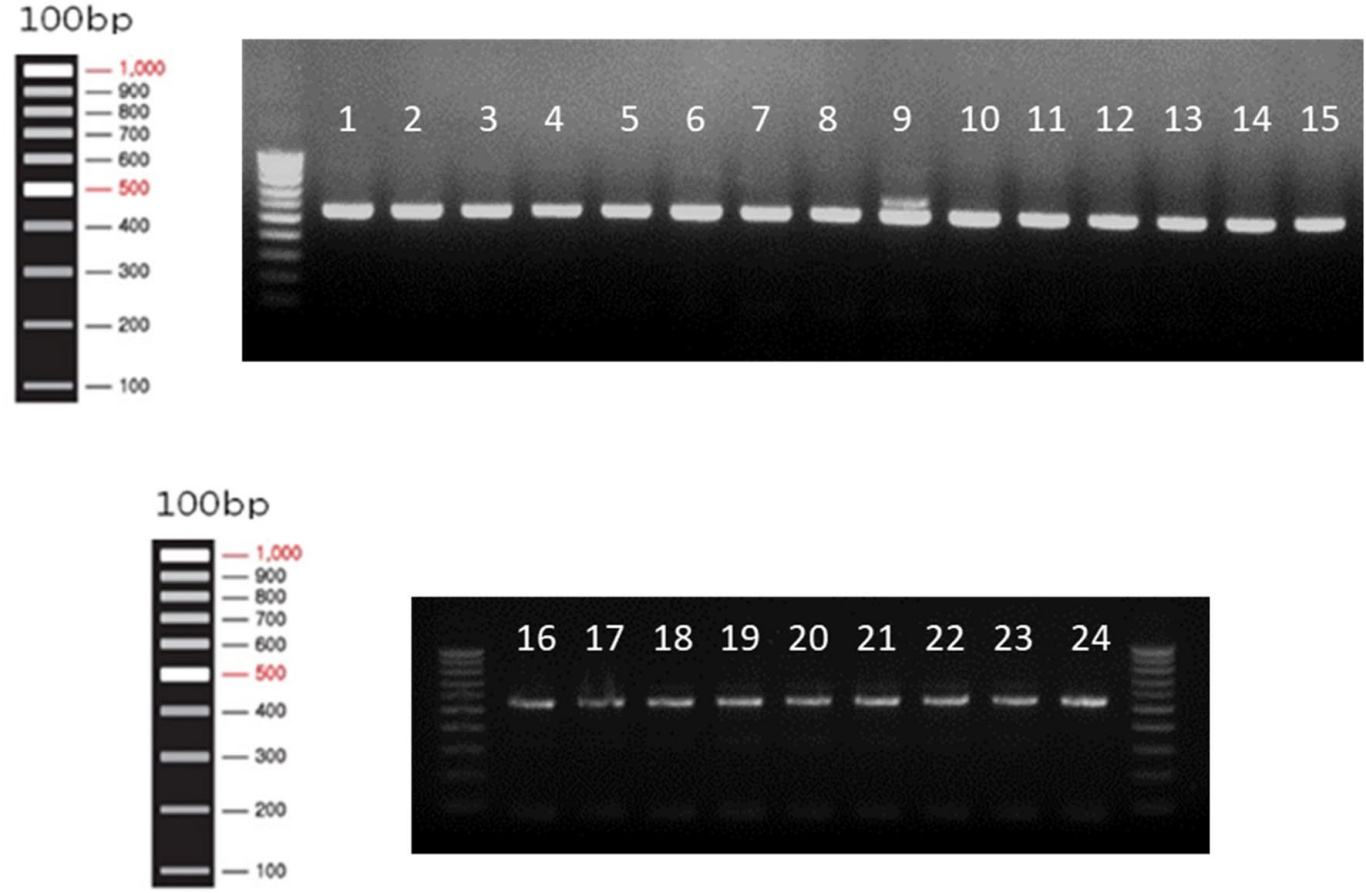
Agarose gel electrophoresis of HSP70 gene. Lane 1–24 are standard for CC type except lane 9. Lane 9 is standard for TC type.

The standard of the HSP70 genotype was shown in Fig. 2, in which CC types were expressed as lanes 1–24 except lane 9 (TC type). Forty calves were selected among forty-six calves based on HSP70 genotyping (CC type: n = 43, TC type: n = 3, -- type: n = 0).

### The effects of dietary protein levels on DMI, protein intake, and growth performance under heat stress

Dietary protein levels in each HS treatment increased protein intake (*P* < 0.01), BW gain (*P* < 0.01), and ADG (*P* < 0.01) in all experimental groups compared with that in the basal diet group (Table 5). However, dietary protein levels in each HS treatment decreased DMI (*P* < 0.01) in all experimental groups compared with that in the control (Table 5). Increased dietary protein levels enhanced protein intake (*P* < 0.01; LP: 506.7 g/d, MP: 607.1 g/d, HP: 697.1 g/d, SEM: 11.8 g/d), BW gain (*P* < 0.01; LP: 15.9 kg, MP: 19.6 kg, HP: 21.8 kg, SEM: 1.3 kg), and ADG (*P* < 0.01; LP: 0.57 kg, MP: 0.70 kg, HP: 0.78 kg, SEM: 0.05 kg) without affecting the DMI (*P* > 0.1; LP: 4.0 kg/d, MP: 4.1 kg/d, HP: 4.1 kg/d, SEM: 0.1 kg/d) (Table 5). HS levels decreased DMI (*P* < 0.01; mild: 4.4 kg/d, moderate: 4.1 kg/d, severe: 3.8 kg/d, SEM: 0.1 kg/d), protein intake (*P* < 0.01; mild: 622.4 g/d, moderate: 614.8 g/d, severe: 573.7 g/d, SEM: 25.5 g/d), BW gain (*P* < 0.05; mild: 21.9 kg, moderate: 19.1 kg, severe: 16.3 kg, SEM: 1.4 kg), and ADG (*P* < 0.05; mild: 0.78 kg, moderate: 0.68 kg, severe: 0.58 kg, SEM: 0.05 kg) (Table 5). No significant interaction (*P* > 0.1) was observed between dietary protein levels and HS levels on growth performance; therefore, the obtained results are presented according to the main effects.

**Table 5:** Effects of dietary protein levels on DMI, protein intake, and growth performance in Korean native beef calves under heat stress.

Item	Threshold	Mild			Moderate			Severe			*P* values for contrast			
	Bas	LP	MP	HP	LP	MP	HP	LP	MP	HP	vs Bas	Protein	HS	Protein * HS
DMI, kg/d	5.3 ± 0.4	4.3 ± 0.2	4.4 ± 0.1	4.5 ± 0.4	4.2 ± 0.2	4.1 ± 0.1	4.1 ± 0.2	3.7 ± 0.2	3.8 ± 0.2	3.9 ± 0.3	< 0.01	0.84	< 0.01	0.89
Protein intake, g/d	635.4 ± 12.14	519.0 ± 9.4	624.5 ± 12.1	723.6 ± 19.9	517.1 ± 12.5	617.6 ± 23.5	709.6 ± 23.8	484.1 ± 11.6	579.1 ± 26.6	658.0 ± 20.7	< 0.01	< 0.01	< 0.01	0.95
Initial BW, kg	205.1 ± 18.5	200.6 ± 13.7	200.6 ± 12.5	204.0 ± 11.2	195.2 ± 10.3	191.3 ± 8.2	196.2 ± 10.1	207.1 ± 8.9	209.6 ± 5.2	212.0 ± 9.8	0.95	0.90	0.21	0.99
Final BW, kg	227.7 ± 17.4	221.1 ± 14.9	222.8 ± 10.8	226.8 ± 11.4	209.9 ± 11.2	212.3 ± 7.0	217.8 ± 10.7	219.8 ± 11.6	225.3 ± 7.2	232.8 ± 9.2	0.94	0.60	0.32	0.99
BW Gain, kg	22.6 ± 1.3	20.5 ± 1.5	22.2 ± 3.4	22.8 ± 1.9	14.6 ± 1.6	21.0 ± 1.5	21.6 ± 0.7	12.6 ± 3.1	15.6 ± 2.4	20.8 ± 0.8	< 0.01	< 0.01	< 0.05	0.52
ADG, kg	0.81 ± 0.05	0.73 ± 0.06	0.79 ± 0.12	0.82 ± 0.07	0.52 ± 0.06	0.75 ± 0.05	0.77 ± 0.03	0.45 ± 0.11	0.56 ± 0.09	0.74 ± 0.03	< 0 .01	< 0.01	< 0.05	0.53

vs Bas = Control (with 12.5% dietary protein levels, THI 68 to 70) vs 9 experimental diets (with 3 dietary protein levels, with 3 THI levels).

THI = Temperature humidity index, THI = (1.8 × Tdb + 32) – [(0.55 – 0.0055 × RH) × (1.8 × Tdb − 26.8).

Threshold = THI 70 to 73, Mild = THI 74 to 76, Moderate = THI 81 to 83, Severe = THI 89 to 91.

LP = Low protein (12.5%), MP = Medium protein (15%), HP = High protein (17.5%).

The data are presented as the means ± standards error (n = 4/group).

### The effects of dietary protein levels on physiological parameters under heat stress

The HR and RT were not changed (*P* > 0.05) by the dietary protein or HS levels during the TN period. However, during the HS period, HR (*P* < 0.01; mild: 73.4 bpm, moderate: 78.6 bpm, severe: 86.3 bpm, SEM: 2.0 bpm) and RT (*P* < 0.01; mild: 39.3 °C, moderate: 39.7 °C, severe: 39.9 °C, SEM: 0.1 °C) were increased as HS level increased (Table 6). Dietary protein levels did not affect (*P* > 0.05) HR (LP: 79.8 bpm, MP: 80.8 bpm, HP: 77.8 bpm, SEM: 2.5 bpm) and RT (LP: 39.6 °C, MP: 39.6 °C, HP: 39.7 °C, SEM: 0.1 (Table 6).

**Table 6:** Effects of dietary protein levels on heart rate and rectal temperature in Korean native beef calves under heat stress.

	Threshold	Mild			Moderate			Severe			*P* values for contrast			
	Bas	LP	MP	HP	LP	MP	HP	LP	MP	HP	vs Bas	Protein	HS	Protein * HS
**HR**														
TN	66.3 ± 2.9	66.8 ± 2.3	70.5 ± 3.2	66.8 ± 2.3	65.0 ± 3.0	64.8 ± 3.3	69.3 ± 7.5	64.8 ± 2.7	68.0 ± 3.2	63.3 ± 3.7	0.79	0.63	0.53	0.54
HS	69.5 ± 3.4	74.0 ± 0.8	74.3 ± 2.7	72.0 ± 4.4	77.8 ± 3.1	80.8 ± 3.3	77.3 ± 4.7	87.5 ± 2.9	87.3 ± 4.4	84.3 ± 4.8	< 0.05	0.62	< 0.01	0.99
**RT**														
TN	39.0 ± 0.1	38.8 ± 0.1	39.0 ± 0.1	38.9 ± 0.1	39.0 ± 0.1	38.9 ± 0.2	38.8 ± 0.1	38.9 ± 0.1	38.9 ± 0.1	39.0 ± 0.1	0.83	0.76	0.91	0.53
HS	39.0 ± 0.1	39.3 ± 0.1	39.3 ± 0.1	39.3 ± 0.1	39.6 ± 0.1	39.7 ± 0.1	39.7 ± 0.0	39.8 ± 0.1	40.0 ± 0.1	40.0 ± 0.1	< 0.01	0.26	< 0.01	0.56

vs Bas = Control (with 12.5% dietary protein levels, THI 68 to 70) vs 9 experimental diets (with 3 dietary protein levels, with 3 THI levels).

THI = Temperature humidity index, THI = (1.8 × Tdb + 32) – [(0.55 – 0.0055 × RH) × (1.8 × Tdb − 26.8).

Threshold = THI 70 to 73, Mild = THI 74 to 76, Moderate = THI 81 to 83, Severe = THI 89 to 91.

LP = Low protein (12.5%), MP = Medium protein (15%), HP = High protein (17.5%).

The data are presented as the means ± standards error (n = 4/group).

### The effects of dietary protein levels on blood parameters under heat stress

During the TN period, no difference (*P* > 0.05) was found in blood cortisol, BUN, glucose, and NEFA (Table 7). However, during the HS period, dietary protein levels in each HS treatment increased cortisol (*P* < 0.01) and BUN (*P* < 0.01) and decreased glucose (*P* < 0.01) and NEFA (*P* < 0.01) in all experimental groups compared with that in the basal diet group (Table 7). Increased dietary protein levels rose glucose (*P* < 0.01; LP: 54.6 ng/mL, MP: 52.7 ng/mL, HP: 63.2 ng/mL, SEM: 3.4 ng/mL) and NEFA (*P* < 0.01; LP: 153.7 µEq/L, MP: 161.1 µEq/L, HP: 178.8 µEq/L, SEM: 25.6 µEq/L) (Table 7). Blood cortisol (*P* < 0.01; mild: 9.6 ng/mL, moderate: 9.3 ng/mL, severe: 18.6 ng/mL, SEM: 1.3 ng/mL), BUN (*P* < 0.05; mild: 14.7 µg/mL, moderate: 18.2 µg/mL, severe: 22.2 µg/mL, SEM: 0.5 µg/mL) increased as HS level increases but decreased glucose (*P* < 0.01; mild: 65.2 ng/mL, moderate: 59.1 ng/mL, severe: 46.2 ng/mL, SEM: 2.7 ng/mL) and NEFA (*P* < 0.01; mild: 182.5 µEq/L, moderate: 180.2 µEq/L, severe: 147.9 µEq/L, SEM: 10.6 µEq/L) (Table 7). There were interactions between dietary protein levels and HS levels in blood glucose level (*P* = 0.07; tendency) (Table 7).

**Table 7:** Effects of dietary protein levels on blood parameters in Korean native beef calves under heat stress.

	Threshold	Mild			Moderate			Severe			*P* values for contrast			
	Bas	LP	MP	HP	LP	MP	HP	LP	MP	HP	vs Bas	Protein	HS	Protein * HS
**Cortisol (ng/mL)**														
TN	8.2 ± 1.0	11.1 ± 1.3	8.8 ± 1.7	8.6 ± 1.4	9.2 ± 1.8	8.7 ± 2.1	8.7 ± 2.7	10.1 ± 1.6	8.6 ± 2.2	9.7 ± 1.6	0.98	0.62	0.89	0.97
HS	9.2 ± 1.3	9.2 ± 1.1	9.8 ± 2.3	9.8 ± 1.8	10.1 ± 1.6	8.4 ± 2.2	9.3 ± 1.8	16.8 ± 1.6	22.3 ± 2.1	16.8 ± 4.4	< 0.01	0.64	< 0.01	0.52
**BUN (μg/mL)**														
TN	16.7 ± 1.2	14.8 ± 0.9	14.2 ± 0.7	13.5 ± 0.8	12.8 ± 0.8	13.2 ± 0.7	14.2 ± 0.9	15.1 ± 0.9	14.7 ± 0.8	13.8 ± 1.2	0.90	0.92	0.91	0.94
HS	17.2 ± 0.8	13.9 ± 0.8	15.7 ± 1.4	14.2 ± 0.9	18.1 ± 0.7	18.8 ± 0.8	17.9 ± 0.8	21.8 ± 0.7	22.4 ± 0.5	22.5 ± 0.7	< 0.01	0.93	< 0.05	0.84
**Glucose (μg/mL)**														
TN	66.5 ± 2.2	65.3 ± 2.1	64.8 ± 2.6	68.8 ± 4.4	63.8 ± 2.3	65.0 ± 3.1	64.0 ± 2.9	66.3 ± 3.0	64.3 ± 6.4	65.0 ± 3.1	0.99	0.82	0.42	0.87
HS	68.5 ± 1.7	66.5 ± 2.2	63.8 ± 2.6	65.3 ± 3.2	59.0 ± 2.6	53.5 ± 4.3	64.8 ± 3.0	38.3 ± 4.7	40.8 ± 6.4	59.5 ± 4.0	< 0.01	< 0.01	< 0.01	0.07
**NEFA (uEq/L)**														
TN	204.2 ± 35.2	227 ± 18.1	176.7 ± 28.7	189.5 ± 32.1	157.6 ± 42.1	210.4 ± 44.7	182.1 ± 24.1	162.7 ± 18.2	191.7 ± 18.1	221.1 ± 18.1	0.98	0.91	0.79	0.94
HS	220.5 ± 28.7	200.5 ± 17.5	168.7 ± 20.8	178.3 ± 19.2	165.4 ± 18.7	185.6 ± 24.3	189.8 ± 7.0	125.3 ± 19.8	162 ± 17.8	156.5 ± 20.0	< 0.01	< 0.01	< 0.01	0.19

vs Bas = Control (with 12.5% dietary protein levels, THI 68 to 70) vs 9 experimental diets (with 3 dietary protein levels, with 3 THI levels).

THI = Temperature humidity index, THI = (1.8 × Tdb + 32) – [(0.55 – 0.0055 × RH) × (1.8 × Tdb − 26.8).

Threshold = THI 70 to 73, Mild = THI 74 to 76, Moderate = THI 81 to 83, Severe = THI 89 to 91.

LP = Low protein (12.5%), MP = Medium protein (15%), HP = High protein (17.5%).

The data are presented as the means ± standards error (n = 4/group).

Supplement of different dietary protein levels decreased lysine (*P* < 0.05), glutamic acid (*P* < 0.05), valine (*P* < 0.05), and histidine (*P* < 0.05) in all experimental groups compared with that in the basal diet group during HS (Table 8). Increased dietary protein levels enhanced lysine (*P* < 0.05; LP: 74.2 nmol, MP: 73.8 nmol, HP: 83.8 nmol, SEM: 5.2 nmol), and glutamic acid (*P* < 0.05; LP: 205.4 nmol, MP: 225.2 nmol, HP: 241.8 nmol, SEM: 19.8 nmol). Increased level of HS decreased lysine (*P* < 0.05; mild: 80.7 nmol, moderate: 78.2 nmol, severe: 73.8 nmol, SEM: 7.2 nmol), glutamic acid (*P* < 0.05; mild: 230.7 nmol, moderate: 232.2 nmol, severe: 207.2 nmol, SEM: 31.2 nmol), valine (*P* < 0.01; mild: 14.7 nmol, moderate: 12.7 nmol, severe: 11.2 nmol, SEM: 0.9 nmol), and histidine (*P* < 0.05; mild: 51.7 nmol, moderate: 50.9 nmol, severe: 43.8 nmol, SEM: 7.5 nmol) (Table 8). There were interactions between dietary protein levels and HS levels in lysine (*P* < 0.05) and valine (*P* = 0.08; tendency) (Table 8).

**Table 8:** Effects of dietary protein levels on blood amino acid profiles in Korean native beef calves under heat stress.

	Threshold	Mild			Moderate			Severe			*P* values for contrast			
	Bas	LP	MP	HP	LP	MP	HP	LP	MP	HP	vs Bas	Protein	HS	Protein*HS
**A.A in plasma (nmol)**														
Lysine	84.3 ± 8.2	80.9 ± 6.5	75.5 ± 6.2	82.5 ± 7.5	78.4 ± 4.2	74.4 ± 2.8	85.5 ± 1.8	67.5 ± 4.7	72.5 ± 7.1	83.5 ± 7.2	< 0.05	< 0.05	< 0.05	< 0.05
Methionine	92.4 ± 10.5	90.8 ± 4.2	85.5 ± 7.2	83.5 ± 7.2	72.5 ± 4.2	74.5 ± 10.1	77.4 ± 7.2	68.5 ± 7.1	65.5 ± 7.2	64.7 ± 4.2	0.09	0.17	< 0.05	0.48
Aspartic acid	22.7 ± 8.2	19.8 ± 4.2	12.5 ± 7.1	22.4 ± 4.1	19.2 ± 7.1	22.4 ± 3.1	19.2 ± 3.2	24.5 ± 3.7	22.4 ± 4.2	25.7 ± 2.2	0.92	0.12	0.62	0.52
Serine	109.1 ± 15.3	102.5 ± 4.1	108.2 ± 9.2	121.1 ± 5.6	122.4 ± 7.2	125.1 ± 7.1	122.1 ± 3.2	125.4 ± 7.1	129.1 ± 7.2	122.1 ± 7.2	0.62	0.84	0.62	0.83
Glutamic acid	242.5 ± 28.4	228.4 ± 7.2	220.4 ± 8.2	246.7 ± 15.8	200.4 ± 7.2	250.4 ± 7.1	252.3 ± 6.2	192.4 ± 7.1	202.4 ± 2.7	220.4 ± 7.1	< 0.05	< 0.05	< 0.05	0.71
Glycine	298.1 ± 19.5	252.4 ± 18.4	262.7 ± 7.2	272.4 ± 3.5	267.4 ± 7.2	292.7 ± 6.2	301.4 ± 23.8	261.5 ± 7.2	292.7 ± 6.3	322.7 ± 6.8	0.42	0.32	0.43	0.71
Alanine	288.4 ± 17.6	252.7 ± 6.2	262.7 ± 9.2	277.4 ± 6.2	222.4 ± 9.2	262.4 ± 7.2	267.4 ± 7.7	222.7 ± 6.2	228.2 ± 9.2	242.7 ± 9.2	0.85	0.97	0.52	0.52
Valine	16.8 ± 2.1	14.2 ± 3.4	13.8 ± 4.6	15.7 ± 6.2	10.2 ± 5.4	13.8 ± 7.1	12.2 ± 6.2	8.6 ± 4.3	10.8 ± 4.1	14.6 ± 6.2	< 0.05	0.07	< 0.01	0.08
Isoleucine	25.2 ± 2.9	23.5 ± 4.9	25.7 ± 9.2	30.4 ± 6.2	29.7 ± 6.2	28.8 ± 6.7	28.7 ± 10.2	34.7 ± 6.2	32.7 ± 1.2	42.7 ± 4.3	0.87	0.76	0.68	0.85
Leucine	32.5 ± 4.2	27.5 ± 6.2	32.4 ± 7.1	33.4 ± 7.2	29.2 ± 6.2	35.7 ± 6.8	42.4 ± 5.5	28.4 ± 6.2	32.7 ± 8.1	42.4 ± 6.2	0.08	0.06	< 0.02	0.32
Tyrosine	21.5 ± 7.2	20.9 ± 6.2	18.7 ± 6.2	15.4 ± 6.2	22.8 ± 6.7	21.9 ± 6.4	18.4 ± 2.5	19.4 ± 3.2	15.4 ± 6.1	12.7 ± 6.2	0.71	0.82	0.90	0.82
Phenylalanine	90.8 ± 10.8	82.4 ± 6.2	84.5 ± 9.1	89.4 ± 4.2	85.4 ± 6.8	92.4 ± 7.1	95.7 ± 6.2	82.7 ± 6.2	92.7 ± 7.4	105.4 ± 6.2	0.81	0.82	0.46	0.83
Histidine	58.5 ± 4.6	48.7 ± 6.2	52.5 ± 6.7	56.8 ± 6.2	45.7 ± 5.2	58.8 ± 1.7	55.7 ± 4.8	42.7 ± 8.4	45.7 ± 6.2	45.2 ± 2.2	< 0.05	0.09	< 0.04	0.73
Arginine	100.8 ± 7.2	95.7 ± 16.4	88.7 ± 6.4	98.1 ± 6.8	92.4 ± 6.8	98.7 ± 6.8	92.4 ± 13.5	90.5 ± 6.2	101.4 ± 8.4	108.7 ± 6.8	0.82	0.85	0.62	0.82
Tryptophan	12.5 ± 1.1	11.8 ± 6.2	9.7 ± 6.2	8.5 ± 0.9	10.9 ± 6.4	14.8 ± 6.2	12.7 ± 6.2	14.5 ± 6.2	11.4 ± 2.9	12.8 ± 6.2	0.24	0.46	0.62	0.66
Cystine	39.4 ± 8.2	32.7 ± 6.1	29.7 ± 6.4	28.4 ± 6.2	32.7 ± 2.8	29.8 ± 4.2	25.7 ± 2.5	28.7 ± 6.1	26.4 ± 1.4	22.4 ± 4.2	0.57	0.21	0.69	0.67

vs Bas = Control (with 12.5% dietary protein levels, THI 68 to 70) vs 9 experimental diets (with 3 dietary protein levels, with 3 THI levels).

THI = Temperature humidity index.

Threshold = THI 70 to 73, Mild = THI 74 to 76, Moderate = THI 81 to 83, Severe = THI 89 to 91.

LP = Low protein (12.5%), MP = Medium protein (15%), HP = High protein (17.5%).

The data are presented as the means ± standards error (n = 4/group).

### The effects of dietary protein levels on HSP70 mRNA expression in PBMCs and hair follicles under heat stress

**Figure 3:**
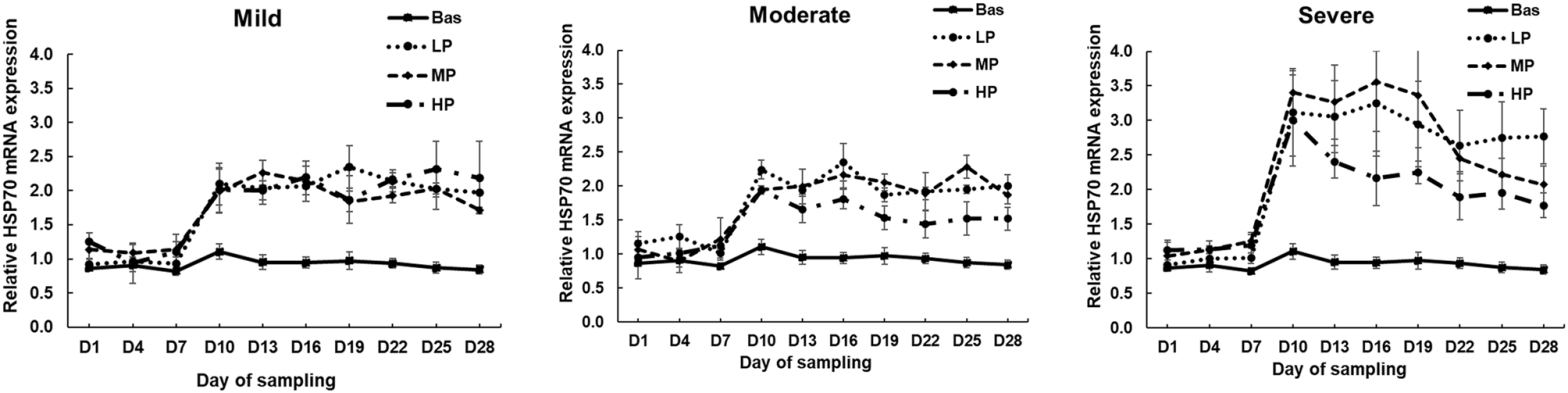
Effects of dietary protein levels on the mRNA expression of HSP70 in the PBMCs of calves during the climate chamber experiment. The data are presented as the means ± standards error (n = 4/group) (protein: *P* = 0.08, HS: *P* < 0.01, day: *P* < 0.01). Threshold = THI 70 to 73, Mild = THI 74 to 76, Moderate = THI 81 to 83, Severe = THI 89 to 91. LP = Low protein (12.5%), MP = Medium protein (15%), HP = High protein (17.5%).

**Figure 4:**
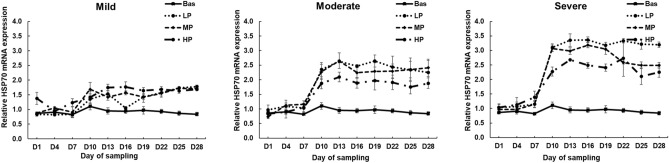
Effects of dietary protein levels on the mRNA expression of HSP70 in hair follicles of calves during the climate chamber experiment. The data are presented as the means ± standards error (n = 4/group) (protein: *P* < 0.05, HS: *P* < 0.01, day: *P* < 0.01). Threshold = THI 70 to 73, Mild = THI 74 to 76, Moderate = THI 81 to 83, Severe = THI 89 to 91. LP = Low protein (12.5%), MP = Medium protein (15%), HP = High protein (17.5%).

The HSP70 gene expression in PBMCs and hair follicles were evaluated by time course over the total period (28 days: Figs. 3, 4). In PBMC, increased dietary protein levels reduced HSP70 gene expression (*P* = 0.08; tendency) (Fig. 3). HS levels increased HSP70 gene expression (*P* < 0.01) (Fig. 3). HSP70 gene expression was increased in LP, MP, and HP groups in each HS levels after rapid exposure to HS conditions by the day of sampling (*P* < 0.01) (Fig. 3). No significant interaction (*P* > 0.1) was observed between dietary protein levels, HS levels and day of sampling on HSP70 gene expression; therefore, the results are presented according to the main effects.

In hair follicles, increased dietary protein levels decreased HSP70 gene expression (*P* < 0.05) (Fig. 4). HSP70 gene expression increased (*P* < 0.01) by increasing the HS level (Fig. 4). HSP70 gene expression was increased in LP, MP, and HP groups in each HS levels after rapid exposure to HS conditions by the day of sampling (*P* < 0.01) (Fig. 4). No significant interaction (*P* > 0.1) was observed between dietary protein levels, HS levels and day of sampling on HSP70 gene expression; therefore, the results are presented according to the main effects.

## Discussion

Climate change causes increased Earth's average yearly surface temperature, and it is inextricably linked with animal agriculture. It has been reported that a drastic rise in temperature and humidity may affect animal's physiology and productivity in a direct or indirect manner. Among others, the number of studies conducted in cattle reported the effects of HS.

In previous dairy cow studies, high-yield cows started to reduce milk yield at a THI of approximately 68^16^. Dairy cows exposed to climate above the threshold THI (mean THI = 82.4) showed hyperthermia, tachypnea, and a significant reduction in DMI^27^. In calves, HS also reduced DMI and ADG^21,28^. Similar to the findings of previous studies, our current results showed that the beef calves exposed to HS decreased DMI and ADG. Reduced feed intake is a conserved response among animals that received HS and is likely an attempt to reduce metabolic heat production^9,12^.

However, we observed that 17.5% (HP) of dietary protein level did not affect DMI but increased protein intake, BW gain, and ADG under HS conditions. There are several nutritional strategies to consider in cattle while under HS.

Feeding energy and nutrient-dense diet, e.g., reduced fiber, increased supplemental fat, and AA, is commonly used to compensate for the reduced feed intake under HS^29^. Primarily, protein is one of the most critical nutrients offered during HS conditions to calves^13^. Protein is required daily for young animals to maintain biological processes, such as repairing tissues and producing blood. Especially in growing calves, protein is one of the most critical nutrients for muscle growth and development^7,13^. Bunting et al.^30^ stated that increased percentage of RUP in the diet improved the growth of HS calves although supplemental fat had no effect on growth performance in summer.

In previous studies, the effects of supplying protein and improving protein solubility are controversial^31,32^. Baumgard and Rhoads Jr^33^ stated that appropriate dietary protein improved growth performance in calves, but the excess dietary protein harmed energy cost. Excess N above requirements reduces ME by 7.2 kcal/g of N^34^. When 19 and 23% of CP diets were fed, milk yield was reduced by over 1.4 kg^35^. Also, it is reported that the milk production did not increase in the moderate CP (16.1%) fed compared with the low CP (12.5%) even though improved plasma glucose concentrations and carbohydrate metabolism in dairy cows under HS^36^. Wildman et al.^37^ stated that high dietary protein (17%) during HS had no effect on DMI and milk production. The energy cost associated with synthesizing and excreting urea accounted for the reduced milk yield^38^. Dietary protein degradability may be especially important under HS conditions. A possible reason why highly degradable protein diets may be harmful during HS was reduced rumen motility and passage rate^29^. This allows for a longer residence time and thus more extensive protein degradation.

Also, diets with low (31.2% of CP) and high (39.2% of CP) RUP fed during HS had no effect on DMI; however, milk yield increased by 2.4 kg/d and BUN reduced from 17.5 to 13.3 mg/100 mL for the diet containing higher RUP^39^. In the current study, heat-stressed calves increased BUN levels. The mechanism of BUN increment is unclear, but this might be due to exceeding rumen ammonia production or protein degradation from skeletal muscle^40,41^. Exceeding ammonia production can be harmful to ruminants and requires extra energy to synthesize urea or egest (7.2 kcal/g of nitrogen; and this increases heat production^34^. However, the protein supplements in this study did not affect the BUN level of calves throughout the trial. Our result is consistent with previous report that revealed additional protein (17.5%) supplements under high ambient temperature can be beneficial to maintain skeletal muscle growth without adverse effect^30^.

It has been previously reported that the blood parameters can be affected by HS. Foremost, HS increased the blood level of fight or flight lipophilic steroid called cortisol throughout the trial. In multiple studies, the correlation between HS and blood cortisol levels were observed^20,28,42^.

In the current study, blood glucose and NEFA decreased in HS groups. This might be related to the negative energy balance resulting from the decreased DMI. This was agreed with previous findings in the rat^43^, chicken^44^, sheep^45^, and cows^46^. A possible scenario that could explain decreased blood glucose level under HS is that of increased glucose utilization as an anti-stress response. Insufficient glucose supplements because of reduced DMI and increased cell glucose uptake could be another reason for decreased blood glucose^34^.

HS condition also appears to influence the lipogenic activity in the animal body. Increased blood NEFA level is typically observed in restricted-fed animals when using lipid as an energy source. A rise in the concentration of circulating NEFAs elevates fatty acid oxidation, thereby maintaining the animal body's glucose level^33^. Post-absorbable carbohydrate metabolism is also altered by reduced insulin action during the dietary restriction, reducing glucose absorption by adipose tissue^7^. The reduced absorption of nutrients coupled with the prolonged net release of NEFA by adipose tissue is a key homeostatic mechanism implemented by malnutrition animals^47^ that can occur due to HS as a consequence of decreased feed intake.

Despite the fact that the blood NEFA level is regulated by energy availability, multiple studies previously noted reduced plasma NEFA level under heat-stressed conditions in rodents^48^, pigs^49^, sheep^50^, and cattle^27,51^ while overall DMI was reduced. This might be related to ketone metabolism. In cattle, high-temperature conditions adversely affect lipogenesis that mobilizes adipose tissue and reduce ketone body synthesis through NEFA, which results in increased reliance on glucose utilization in the body^52^. Also, Baumgard and Rhoads Jr^33^. suggested that this is a natural mechanism for withstanding heat loads, as *β*-oxidation of NEFA can generate more metabolic heat than that of carbohydrates.

Moreover, HS increased lipoprotein lipase in adipose tissue^48^, suggesting that adipose tissue of hyperthermia animals has an increased capacity to absorb and store intestinal and hepatic-derived triglycerides^33^.

In the current study, heat-stressed animals given 17.5% of dietary protein levels (HP) tended to increase blood glucose and NEFA. This may indicate that the HP was used as an energy source to synthesize from glucose and NEFA^33^.

Changes in postabsorptive nutrient partitioning support a controlling physiological state. A well-described mechanism is a glucose-sparing effect is that growing animals utilize fat or protein source when on a lowered plane of nutrition or in a negative balance. Inadequate nutrient consumption during HS conditions is associated with various metabolic changes implemented to support the synthesis of high-priority tissues like a skeletal muscle^53^.

We also noted that the blood contents of lysine, glutamic acid, valine, and histidine are decreased as HS is severe. When plasma glucose level is low, AA utilization from gluconeogenesis is observed in heat-stressed cows^54^. Additionally, we assumed that reduced blood flow might also be suppressed the absorption of AAs. Heat stress alters the nutrient partitioning, leading to reducing the delivery of protein precursors to the adipose tissue or muscle^34^. In the same context, cows under HS reduced plasma free AA, and increased BUN, MUN, and UUN, which combined with similar microbial crude protein synthesis and decreased milk protein output, suggesting increased whole-body AAs utilization^27^. Previous studies revealed that the contribution of AAs to the circulating glucose pool is increased during the HS situation^27,55^. 78% of plasma AAs reduction was due to the decline in gluconeogenic AAs^27^. This study suggests that the plasma AAs decline during HS may result from increased AAs utilization for gluconeogenesis. We found that HP increased lysine and glutamic acid in heat-stressed Korean native beef calves in the current study.

In cattle, lysine is inadequately synthesized in the body; therefore, AA needs to be provided by the diet to fulfill the requirement. In addition, glutamic acid is one of the semi-essential AAs in skeletal muscle^56^. Lower lysine and glutamic acid concentration could be related to DMI reduction due to HS. Increased dietary protein led to improved lysine and glutamic acid utilization in animals under HS. It also led to help calves to sustain growth.

Additionally, other amino acids including lysine, methionine, glutamic acid, valine, isoleucine, and leucine were increased in the HP group. Based on our findings, the supplement of a higher protein level is suggested during heat exposure in calves. Additional protein supplements can cause increased glucogenic and ketogenic activity, which can diminish the adverse effects of HS.

The HSP genes result in the expression of proteins with chaperone roles that act in protecting cells from HS and removing damaged proteins^22,57^. Also, HSP70 polymorphisms found in the functional promoter and HSP70 gene has been associated with mRNA stability and stress response in animals. In a previous study, it has been reported that the difference in HSP70 gene expression and cell viability in PBMC under HS conditions depends on HSP70 genotypes (e.g. CC, C-, --) in dairy cows^14^. However, in Korean beef calves used in this study, there are no prior studies that considered the HSP70 genotype related to HS. Therefore, we used same HSP70 genotype to select individuals with genetic traits that have at least the same heat resistance within the treatment group. In the current study, the HSP70 gene expression in PBMCs and hair follicles was increased in HS groups. One of the signals for HSP70 synthesis is protein denaturation in response to elevated temperature^58^. The transcription of HSP genes and, consequently, the synthesis of HSPs are enhanced upon exposure of cells to HS^59^. The hyperthermia resulting from HS can also disturb the cellular functions and result in physiological changes that cause a negative impact on animal production^11^. In cattle, HSP72 is absent or expressed at a low level under non-stressed conditions and is referred to as the inducible member of the HSP70 family^60^. The expression level of HSP70 was reported to increase at the cellular level under HS conditions^61^. The current study showed that HSP70 gene expression in hair follicles increased in all dietary protein level groups (LP, MP, and HP). This can be interpreted as an appropriate dietary protein level (17.5%) that affects the synthesis of HSP70 and enhances immune function. In addition, our current study suggested that the hair follicles can be used as a non-invasive method to demonstrate the level of HSP in HS animals.

## Conclusion

**Figure 5:**
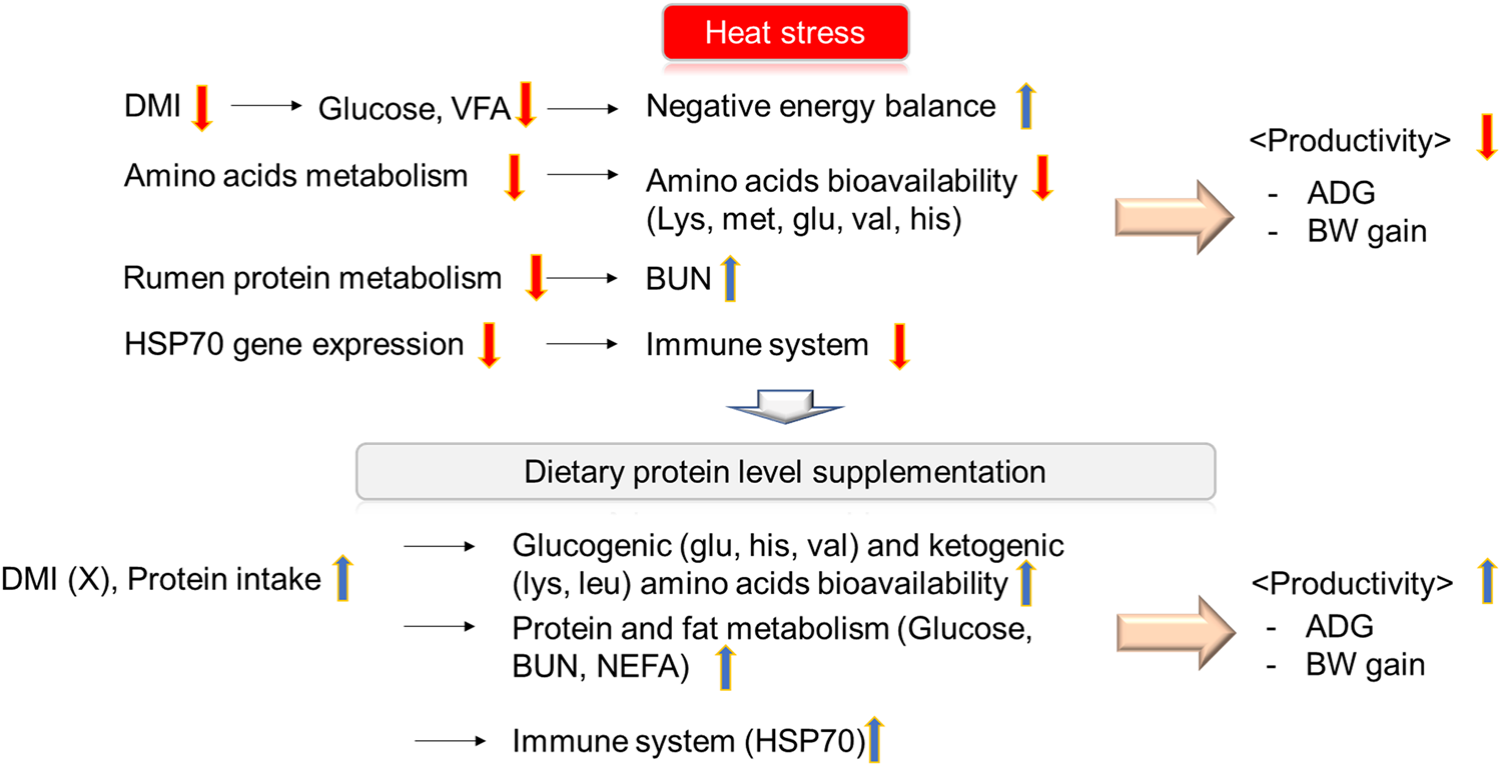
Effects of dietary protein levels on productivity in Korean native beef calves.

Heat stress directly affected protein metabolism and growth, including decreased BW gain and ADG in heat-stressed Korean native beef calves. The decreased DMI cannot wholly define the decline in BW gain and ADG. The decrease in blood glucose, NEFA, and blood AAs profiles and increased BUN and HSP70 gene expression during heat stress affect growth through energy and protein metabolism. In conclusion, the appropriate dietary protein level was considered to be 17.5% due to improved growth performance by energy and protein metabolism, utilization of AAs and the synthesis of HSP70, and enhanced immune function (Fig. 5).

## Abbreviations

HS: Heat stress
LP: Low protein
MP: Medium protein
HP: High protein
THI: Temperature-humidity index
ADG: Average daily gain
HR: Heart rate
RT: Rectal temperature
NEFA: Non-esterified fatty acids
AAs: Amino acids
PBMC: Peripheral blood mononuclear cell
HSP: Heat shock protein
DMI: Dry matter intake
NRC: National Research Council
PCR–RFLP: Polymerase chain reaction-restriction fragment length polymorphism
RH: Relative humidity
CP: Crude protein
TN: Thermoneutral
BW: Body weight
DM: Dry matter
NDF: Neutral detergent fiber
ADF: Acid detergent fiber
AOAC: Association of official analytical chemists
BPM: Beat per minute
BUN: Blood urea nitrogen
PBS: Phosphate buffered saline
DEPC: Diethyl pyrocarbonate
RNA: Ribonucleic acid
PCR: Polymerase chain reaction
DNA: Deoxyribonucleic acid
GAPDH: Glyceraldehyde 3-phosphate dehydrogenase
RPS15A: Ribosomal protein S15a
B2M: Beta 2 microglobulin
EDTA: Ethylenediaminetetraacetic acid
GLM: Generalized linear model
HSD: Honest significant difference
ME: Metabolizable energy
RUP: Rumen undegradable protein
MUN: Milk urea nitrogen
UUN: Urine urea nitrogen
MCP: Microbial crude protein
